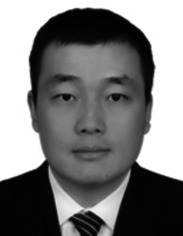# Homogeneous Catalysts Based on First‐Row Transition‐Metals for Electrochemical Water Oxidation

**DOI:** 10.1002/cssc.202001876

**Published:** 2020-10-16

**Authors:** Lu‐Hua Zhang, Simon Mathew, Joeri Hessels, Joost N. H. Reek, Fengshou Yu

**Affiliations:** ^1^ School of Chemical Engineering and Technology Hebei University of Technology Tianjin 300130 P. R. China; ^2^ van't Hoff Institute for Molecular Sciences Universiteit van Amsterdam Science Park 904 1098 XH Amsterdam The Netherlands

**Keywords:** Homogeneous catalysis, Molecular electrochemistry, Oxidation, Transition metals, Water Splitting

## Abstract

Strategies that enable the renewable production of storable fuels (i. e. hydrogen or hydrocarbons) through electrocatalysis continue to generate interest in the scientific community. Of central importance to this pursuit is obtaining the requisite chemical (H^+^) and electronic (e^−^) inputs for fuel‐forming reduction reactions, which can be met sustainably by water oxidation catalysis. Further possibility exists to couple these redox transformations to renewable energy sources (i. e. solar), thus creating a carbon neutral solution for long‐term energy storage. Nature uses a Mn−Ca cluster for water oxidation catalysis via multiple proton‐coupled electron‐transfers (PCETs) with a photogenerated bias to perform this process with TOF 100∼300 s^−1^. Synthetic molecular catalysts that efficiently perform this conversion commonly utilize rare metals (e. g., Ru, Ir), whose low abundance are associated to higher costs and scalability limitations. Inspired by nature‘s use of 1^st^ row transition metal (TM) complexes for water oxidation catalysts (WOCs), attempts to use these abundant metals have been intensively explored but met with limited success. The smaller atomic size of 1^st^ row TM ions lowers its ability to accommodate the oxidative equivalents required in the 4e^−^/4H^+^ water oxidation catalysis process, unlike noble metal catalysts that perform single‐site electrocatalysis at lower overpotentials (*η*). Overcoming the limitations of 1^st^ row TMs requires developing molecular catalysts that exploit biomimetic phenomena – multiple‐metal redox‐cooperativity, PCET and second‐sphere interactions – to lower the overpotential, preorganize substrates and maintain stability. Thus, the ultimate goal of developing efficient, robust and scalable WOCs remains a challenge. This Review provides a summary of previous research works highlighting 1^st^ row TM‐based homogeneous WOCs, catalytic mechanisms, followed by strategies for catalytic activity improvements, before closing with a future outlook for this field.

## Graphical abstract

**First row enhancements**: In this focus Review, the potential of homogeneous catalysts that employ 1^st^ row transition‐metals for electrocatalytic water oxidation are highlighted. A comprehensive summary of the strategies utilized for reactivity enhancement in Fe‐, Cu‐, Ni‐ and Co‐based molecular electrocatalysts reported over the last decade is given prior to presenting a future outlook for this field.

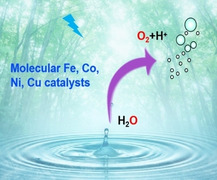

## 1 Introduction

Artificial photosynthesis is a promising approach to solar energy storage in the form of chemical bonds, especially hydrogen and hydrocarbons that can be stored and immediately implemented into existing energy generation infrastructure.^[1]^ Of key importance to this goal is the splitting of water to access the requisite protons (H^+^) and electrons (e^−^) for subsequent reductive chemistry. The overall water splitting reaction [Eq. (1)] is thermodynamically uphill. Artificial photosynthesis is focused on powering this conversion using sustainable energy sources. The water splitting reaction is a redox conversion, where the oxidative half‐reaction, the conversion of H_2_O to O_2_ and H^+^ [Eq. (2)] has a standard potential (*E*_0_) of +1.23 V vs. NHE at pH 0. In this case, the complementary reductive half‐reaction would be the reduction of H^+^ to H_2_ [Eq. (3)], which has a standard potential (*E*_0_) of 0 V vs. NHE at pH 0. However, it is also possible to combine water oxidation

[Eq. (2)] with the reduction of CO_2_ to afford carbon‐based fuels [Eq. 4].

(1) $$2H{}_{2}O{}_{\left(l\right)}\to O{}_{2(g)}+2H{}_{2(g)}$$

(2) $$2H{}_{2}O{}_{\left(l\right)}\to O{}_{2(g)}+4e{}^{-}+4H{}^{+}{}_{\left(aq\right)}$$

(3) $$2H{}^{+}{}_{\left(aq\right)}+2e{}^{-}\to H{}_{2(g)}$$

(4) $$\mathrm{CO}{}_{2(g)}+H{}^{+}{}_{\left(aq\right)}+e{}^{-}\to \mathrm{Carbon}\text{-}\mathrm{based}\;\mathrm{fuels}$$

Thus, the electrons and protons generated by electrocatalytic water oxidation [Eq. (2)] are required for the respective formation of hydrogen [Eq. (3)] or hydrocarbons [Eq. (4)] in the reduction reaction.^[2]^ Beyond being a thermodynamically uphill electrocatalytic transformation, water oxidation catalysis is kinetically sluggish and often requires overpotential because it involves two water molecules and it is a multielectron/multiproton process. This is particularly true for catalysts based on first row transition metal complexes. In this regard, better performing catalysts are inevitably required for future solar fuel applications, with a focus on enhance reaction rate, lower overpotentials and high stability.

From a practical standpoint, WOCs have been studied using two different strategies: reaction driven by chemical oxidation with a sacrificial oxidant or an electrochemical oxidation using a bias voltage. The former method allows for rapid screening of potential WOCs for catalytic activity,^[3]^ but it is limited by the fixed driving force and specific operational pH (i. e. pH <1 with Ce^IV^) of the oxidant.^[4]^ In contrast, electrocatalysis is controllable by tuning both the bias potential and tunable pH. The bias voltage is comprised of the thermodynamically determined potential for water oxidation (i. e. +1.23 V vs. NHE @ pH 7) and an overpotential (*η*).^[5]^ In a practical sense, the driving force can be attenuated by adjusting the applied potential, enabling control of catalysis rates to facilitate the coupling with the proton/CO_2_ reduction half‐reactions upon implementation into a complete fuel cell.

Research on water oxidation electrocatalysis has been progressing at a steady pace both in homogeneous and heterogeneous catalyst development. Heterogeneous catalysts are attractive because of their impressive long‐term stability,^[6]^ however the investigation of homogeneous electrocatalysts continues to attract attention due to inherent opportunities that are inaccessible to heterogeneous catalysts.[^4^, ^7^] These include: 1) homogeneous solutions enable mechanistic studies by several spectroscopic approaches enabling the elucidation of catalytic mechanisms to guide future electrocatalyst development.^[8]^ 2) Molecular structures are well defined and structurally tunable. It is possible to systematically tune catalytic activity via rational ligand design based on mechanistic insights, while electrode immobilization is possible through ligand modification.^[9]^ 3) Homogeneous WOCs exhibit a higher (metal) atom economy and turnover frequency (TOF) than heterogeneous counterparts.^[10]^ For example, a TOF more than 1000 s^−1^ for a pentanuclear iron catalyst has been reported in a homogeneous electrochemical system.^[11]^ The TOF is much higher than those achieved by any heterogeneous metal oxide WOCs (10^−4^–10^1^ s^−1^)[^6^, ^12^] and by the naturally photosystem II (100–300 s^−1^).^[13]^ Homogeneous catalysts have been adapted for electrode attachment and widely implemented in photoanodes,[^9d^, ^14^] Z‐schemes,^[9c]^ and overall water splitting systems.^[15]^ The development of efficient WOCs based on abundant materials is a required step for development of commercially viable solar‐fuel devices.^[7b]^

The most efficient WOCs are based on Ru and Ir, displaying catalytic activity at low overpotentials with high TOF of 10^3^ s^−1^ and stabilities (i. e. turnover number, TON) up to 10^6^.^[16]^ However, the limited availability of these metals introduces cost and scalability limitations that reduce the competitiveness of strategies employing scarce elements. The high prices are caused by the low natural abundance of noble metals, compared to their 1^st^ row counterparts. In addition, the prices of these elements will likely increase with demand, hampering the potential impact of terawatt‐scale renewable fuel generation technology that relies on rare/noble elements.

To counteract this, WOCs based on earth‐abundant, 1^st^ row transition metals (TM) featuring a low overpotential and good stability under mild conditions are highly desirable. However, 1^st^ row TM based WOCs generally require high overpotential to overcome the large reaction barrier caused by the four‐electron transfer process, hindering the overall efficiency of the system.^[2]^ In spite of this, extensive efforts have been dedicated to developing efficient earth‐abundant based WOCs, with numerous catalysts reported.

## 2 Orbital Structure and Catalyst Performance

From a fundamental point‐of‐view, reasons of both thermodynamic and kinetic origin account for the reduced catalytic performance of 1^st^ row TMs compared to their noble metal counterparts, originating from the core‐orbital structure of the metal. Thermodynamically, two arguments are evident; a) the lower association constant of 1^st^ row TMs with ligands and b) the very stable metal‐oxo species (M=O) of 1^st^ row TMs. The associated kinetic argument is that the bond distances of 1^st^ row TMs experience greater perturbation upon redox events, with these larger conformational changes effecting reduced electron transfer rates.

### 2.1 Catalyst thermodynamics of 1^st^ row TM complexes

**Figure 1:**
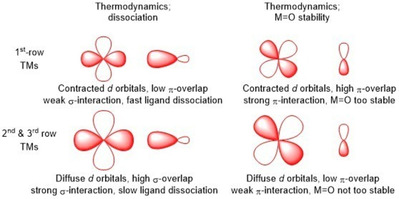
Thermodynamic arguments against 1^st^ row TMs as water oxidation catalysts.

1^st^ row TMs form coordination bonds with 3d orbitals, in contrast to the 4d or 5d orbitals of 2^nd^ and 3^rd^ row TMs, respectively.^[17]^ As 3*d* orbitals are smaller (i. e., contracted), there is less σ‐overlap with donor atoms, causing the association constants of 1^st^ row TM ligands to be substantially lower than those of noble metals (Figure 1), whose d orbitals are larger (i. e., diffuse). This enables a greater frequency of ligand dissociation in 1^st^ row TM catalysts, with free ligands exhibiting a greater propensity toward oxidative degradation. Furthermore, the dissociated metal ion can form metal nanoparticles, preventing reassociation to an intact ligated structure. Both of these pathways lead to a faster deactivation of 1^st^ row TM WOCs. To counteract this, multidentate ligands have be employed, in all (relatively) successful 1^st^ row TM WOCs by employing either tetra‐ or pentadentate ligands.[^4^, ^18^] This is in stark contrast to iridium and ruthenium WOCs where bidentate and even monodentate ligands are often employed.^[19]^

The need for multidentate ligands imposes a synthetic limitation on ligand design, which is further compounded by a reduction in structural flexibility. As water oxidation requires four electrons, several proton transfers and two water molecules, the ligand generally must accommodate the metal in various oxidation states, requiring the complex to access a wider range of geometries. Thus, a more rigid ligand environment will likely lead to greater energy differences between intermediate transition state geometries within the catalytic cycle. For example, flexible donor‐ligands have been employed with great success in ruthenium‐based WOCs, with the [2,2′ : 6′,2′′‐terpyridine]‐6,6′′‐dicarboxylato (tda) ligand published by the group of Llobet reaching very high TOFs (>10^6^ s^−1^) at a relatively low overpotential of 380 mV.^[16c]^ Based on thermodynamics, water splitting requires 1.23 eV, meaning if each of the four electron transfers require exactly 1.23 eV, then there will be no overpotential required for the reaction.^[20]^ However if one step costs less than 1.23 eV, another electron transfer step will have to cost more. If an electron transfer step costs more than 1.23 eV, a higher potential will have to be applied, resulting in an overpotential for the reaction. In 1^st^ row TMs, the metal‐oxo species (M=O) is relatively stable, originating from the high π‐orbital overlap between metal ion and ligand donor‐atoms. In the most commonly proposed water oxidation mechanisms, this M=O species is a part of the catalytic cycle and can be formed by oxidizing water by two electrons.[^8d^, ^21^] If this M=O species is relatively stable, the first two electron transfers may be very easy to perform (i. e.,≪1.23 eV) and consequently, thermodynamics dictates that the following steps in the mechanism will require more energy (i. e.,≫1.23 eV). Therefore, 1^st^ row TMs will generally have a relatively high overpotential for water oxidation compared to 2^nd^ and 3^rd^ row metals (Figure 1).

### 2.2 Catalyst kinetics in 1^st^ row TM complexes

**Figure 2:**
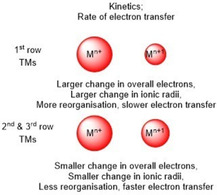
Kinetic arguments against 1^st^ row TMs as water oxidation catalysts.

There is also a kinetic argument as to why 2^nd^ and 3^rd^ row TMs perform better than their 1^st^ row counter parts. A change in the oxidation state of a metal within a complex will affect the bonding distances to any surrounding ligands.^[17]^ As 1^st^ row TMs have less electrons than 2^nd^ and 3^rd^ row counterparts, their net number of electrons experience a greater relative change upon metal‐centered redox events. Consequently, a 1^st^ row TM will experience a greater change in ionic radius upon a redox event (Figure 2).

In electrochemistry, the rate of electron transfer is strongly influenced by the reorganization energy of the redox active species upon reduction or oxidation.^[22]^ Thus, as 1^st^ row TM electrocatalysts experience larger geometrical changes during redox events than 2^nd^ and 3^rd^ row TMs, the electron transfer rates associated with them will be slower. As WOCs need to transfer four electrons, they generally need to accommodate several redox states. This means that, for 1^st^ row TMs performing water oxidation, the accompanying oxidation reactions will generally have lower rates than their 2^nd^ and 3^rd^ row TM counterparts (Figure 2). Importantly, if an oxidation step is rate limiting in the catalytic cycle for a certain system, this will lead to lower rates.

In nature, manganese is utilized within the active site of the Mn_4_Ca cluster located in the oxygen evolution center of green plants.^[23]^ Inspired by nature, Mn complexes were initially explored to develop earth‐abundant WOCs. However, in recent years reports of electrochemical WOCs based on Mn are limited and they were reviewed elsewhere.^[18a]^ As the bulk of progress has been made with iron, copper, nickel, and cobalt complexes, we have written this review article to summarize the recent progress in homogeneous water oxidation electrocatalysts based on earth‐abundant 1^st^ row transition metals (i. e., Fe, Cu, Ni Co) over the last decade, followed by an outlook on further WOC design.

### 2.3 Distinguishing between homogeneous and heterogeneous catalysts

The relatively low association constants between 1^st^ row TM and ligands cause a greater frequency of ligand dissociation.^[24]^ During electrocatalysis, free ligands demonstrate a greater propensity toward oxidative degradation, whereas dissociated metal ions can form metal nanoparticles. Nanoparticle formation inhibits reassociation of the free metal ion to regenerate an intact ligated structure,[^12^, ^25^] while also possessing the ability to act as potent water oxidation catalysts under testing conditions. For this reason, it is imperative to identify the nature of the catalytically‐active species during evaluation of 1^st^ row TM based homogeneous catalysts.

Essential detection experiments are centered on post‐electrolysis analyses of the working electrode and the electrolyte. Evaluation of the catalytic performance with the used electrode (after bulk electrolysis) in fresh buffer solution is necessary to confirm the presence and activity of metal‐based nanoparticles that may form upon catalyst degradation. Typically, one evaluates if a larger current is derived from an electrode after bulk electrolysis than for the blank electrode. If the used electrode demonstrates catalytic activity, then the presence of an active species on the electrode surface is implied.^[26]^ The detection of metal‐based nanoparticles on electrodes (after bulk electrolysis) can be achieved spectroscopically by energy‐dispersive X‐ray spectroscopy (EDX) and X‐ray photoelectron spectroscopy (XPS).^[27]^

The complementary scenario exists where a potentially formed insoluble nanoparticle may remain in the electrolyte.^[28]^ Dynamic light scattering (DLS) is a powerful technique to detect and monitor the formation of dispersed nanoparticles in solutions.^[29]^ On the other hand, quantitative comparison of the homogeneous complex concentration before and after electrolysis can be established by UV‐vis spectroscopy. A constant concentration suggests the stable nature of the homogeneous catalyst under electrocatalytic conditions,^[30]^ otherwise further analysis is needed for active species identification. Another strategy is the comparison of catalytic current with and without added metal ions scavengers such as bipyridine^[31]^ and EDTA.^[25]^ These chemicals can capture free metal ions and inhibit metal oxide formation. Therefore, chromoamperometric experiments in with metal ions scavengers present add credence to the molecular nature of a homogeneous WOC. In addition to these typical analyses, other specific detection strategies have also been developed.

Recently, the formation of NiO_*x*_ was confirmed with a homogeneous macrocyclic nickel(II) complex [Ni(meso‐L)](ClO_4_)_2_ (L=5,5,7,12,12,14‐hexamethyl‐1,4,8,11‐tetraazacyclotetradecane) as catalyst by our group.^[32]^ The structure was originally reported as a homogeneous WOC at pH 7, due to instability of the NiO_*x*_ formed during bulk electrolysis. The NiO_*x*_ desorbed rapidly upon removal of applied bias, thus the metal‐based nanoparticle species eluded detection. The use of an electrochemical quartz crystal microbalance (EQCM) enabled the in situ probing of mass increases on the working electrode during bulk electrolysis, implying the deposition of insoluble species. The deposited species was stabilized by tuning the pH to 8.5 immediately after bulk electrolysis, enabling subsequent confirmation of adsorbed NiO_*x*_ by XPS measurements of the working electrode. Thus, to clearly identify whether a transition‐metal based WOC is homogenous or heterogeneous, it requires a combination of the detection techniques mentioned above.

## 3 Homogeneous Iron‐Based WOCs

As the second most abundant metal within the earth's crust, iron is an essential element in a variety of enzymes and it also shows a rich chemistry in synthetic catalysis due to its redox properties.^[33]^ For example, iron has received wide attention in dioxygen activation by invoking high‐valence intermediates,^[34]^ inspiring researchers to broaden the application Fe‐based molecular catalysts to the opposite reaction‐water oxidation. The last decade has demonstrated the ability for Fe‐based molecular catalysts to be active for water oxidation. Costas et al. reported a series of Fe‐based complexes with two free *cis‐* coordination sites which were proved to be efficient WOCs with TON up to 1000 driven by sodium periodate.^[35]^ These catalysts driven by the use of a sacrificial reagent have been reviewed elsewhere by Sun et al.,^[36]^ while within this review we discuss molecular electrocatalysts.

### 3.1 Iron‐based WOCs for electrocatalysis in organic solvents

**Figure 3:**
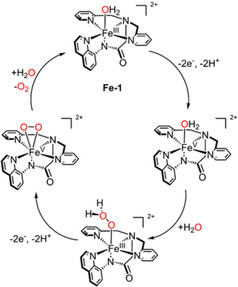
The proposed catalytic mechanism of **Fe‐1** ^[37]^ (*η*=700 mV, TOF=0.15 s^−1^) in propylene carbonate/water.

The research on homogeneous Fe‐WOCs started late compared to other metals, with the first Fe electrochemical WOC [Fe^III^(dpaq)(H_2_O)]^2+^ (**Fe‐1**, dpaq=2‐[bis(pyridin‐2‐ylmethyl)]amino‐*N*‐quinolin‐8‐yl‐acetamido, an octahedral complex reported by Meyer et al. in 2014.^[37]^ As shown in Figure 3, water oxidation was proposed to be mediated by Fe^V^(O)^2+^ generated from Fe^III^(H_2_O)^2+^ through a oxidative 2e^−^/2H^+^ proton coupled electron transfer (PCET) process. Following this, the peroxide Fe^III^(OOH_2_)^2+^ was produced by the reaction of Fe^V^(O)^2+^ with water via a nucleophilic attack, in line with a kinetic rate that is first order in both catalyst and added water. Regeneration of the **Fe‐1** catalyst is possible with O_2_ release occurring with concomitant H_2_O coordination and continuation of the catalytic cycle. Although the Faradaic efficiency is only 45 % after 15 h of bulk electrolysis, the complex demonstrates to be impressively stable. Only minor catalyst decomposition was observed, especially compared to Fe WOCs in acidic solutions which show water oxidation catalysis driven by Ce^IV^ or NaIO_4_ as oxidants.^[38]^ This work opened the door for iron‐based water oxidation electrocatalysts, providing inspiration for future designs of robust Fe‐catalysts.

**Figure 4:**
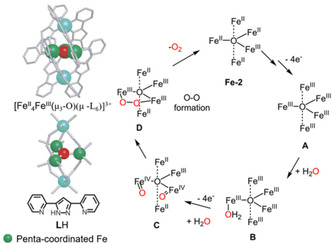
Ball‐and‐stick representations of the molecular structure, the Fe_5_O core structure of [Fe^II^_4_Fe^III^(μ_3_‐O)(μ‐L)_6_]^3+^ (**Fe‐2**, *η*>500 mV, TOF>1000 s^−1^)^[11]^ and the chemical structure of LH (left), deprotonated to L upon catalyst formation. The proposed catalytic mechanism of **Fe‐2** (right). (Copyright 2016 Nature Publishing Group, adapted with permission).

Remarkable progress on Fe‐based water oxidation electrocatalysts was achieved by Okamura *et al*.,^[11]^ with the research of a pentanuclear iron catalyst [Fe^II^_4_Fe^III^(μ_3_‐O)(μ‐L)_6_]^3+^ (**Fe‐2**, LH=3,5‐bis(2‐pyridyl)pyrazole, Figure 4 left). Cyclic voltammetry (CV) measurements reveal five reversible waves at *E*_1/2_=−0.55, 0.13, 0.30, 0.68 and 1.08 V vs. ferrocene/ferrocenium (Fc/Fc^+^), assigned to five distinct one‐electron redox events for each of the iron Fe^III^/Fe^II^ couples demonstrating the ability to accommodate multiple oxidative equivalents to achieve the transformations. In the presence of water, the large irreversible anodic current at ∼1.08 V was assigned to water oxidation process. A TOF value over 10^3^ s^−1^ and a TON of about 10^6^–10^7^ were calculated based on the electrochemical data with the assumption that the thickness of the reaction‐diffusion layer adjacent to the electrode surface (μ) is of the order of *μ*≈$\sqrt{D/k_{cat}}$ . A Faradaic efficiency of 96 % was determined, based on a 4e^−^ process, suggesting excellent selectivity. A catalytic cycle was proposed, depicted in Figure 4, with the first intermediate Fe^III^_5_ (**A**) generated from the initial Fe^II^_4_Fe^III^ (**Fe‐2**) by four distinct 1e^−^ oxidation processes, verified by the unchanged oxidation potentials in CV in the presence of water. Water coordination to **Fe‐2** to yield intermediate **A** preceded the addition of a second water molecule and deprotonation (sequential or simultaneous, not determined) to afford the bis‐oxo species (**C**). Quantum chemical calculations indicate that the mixed‐valence Fe^II^_2_Fe^III^(Fe^IV^=O)_2_ state is the lowest in energy and thus represents the resting state. The rate‐determining step (RDS) is the O−O bond formation from complex **C** to form per‐oxo intermediate (**D**). The high reactivity of this complex is explained by a low activation barrier for this step that occurs without spin‐rearrangement, as a consequence of the co‐facial oxo groups in **C**. From the per‐oxo intermediate **D**, dioxygen is liberated and the catalyst returns to its initial state. The catalytic mechanism was revisited by Liao *et al*., and they found that O−O bond formation was taking place at the Fe^III^_3_(Fe^IV^=O)_2_ state rather than Fe^II^_2_Fe^III^(Fe^IV^=O)_2_ based on the very high energetics for the formation of the latter.^[39]^ Although the activity of **Fe‐2** as a WOC was limited to electrocatalysis in organic solvents, it presents a key step for the development of earth‐abundant WOCs that demonstrates catalytic rates comparable to Ru catalysts.^[16d]^

### 3.2 Water soluble iron‐based WOCs

**Figure 5:**
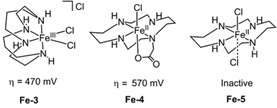
Structure of WOCs **Fe‐3**, **Fe‐4** and **Fe‐5** (pH 7.5 aqueous system).^[40]^

Both **Fe‐1** and **Fe‐2** demonstrate an impressive electrochemical activity (TOF>10^3^ s^−1^) and longevity (TON=10^6^–10^7^), but these complexes also suffer from limitations, including a large required overpotential (>500 mV) and the need for electrocatalysis in organic solvent. To circumvent this, examples of water‐soluble Fe‐based WOCs were explored at the same time. Three water soluble iron complexes (**Fe‐3**, **Fe‐4** and **Fe‐5**, Figure 5) were evaluated for water oxidation by Hetterscheid et al., employing an on‐line electrochemical mass spectrometry strategy to identify the products of electrocatalysis and determine the onset potentials of water oxidation.^[40]^ Oxygen detection at 1.7 V vs. RHE at pH 7.5 with an overpotential of 470 mV established electrocatalysis water oxidation for **Fe‐3** featuring an initial *cis*‐configuration, while trace amounts of CO_2_ was observed from oxidative decomposition of the organic ligand. This behavior is in line with other iron‐based molecular WOCs that have low Faradaic efficiencies or low TON.^[38b]^ The introduction of an axially coordinating carboxylate residue structure in **Fe‐4** caused water oxidation catalysis to occur from 1.8 V vs. RHE, indicating the carboxylic acid group did not have a positive influence, but was not further explored. Meanwhile, no obvious activity was detected for **Fe‐5**, consistent with the conclusions of a preceding report on the water oxidation catalysis of **Fe‐5** with Ce(NH_4_)_2_(NO_3_)_6_ as a sacrificial oxidant.^[38a]^ Importantly, the authors successfully demonstrated the utility and reliability of on‐line mass spectrometry in the determination of the real onset potentials by ruling out side reactions, (e. g. ligand oxidation).[^40^, ^41^]

**Figure 6:**
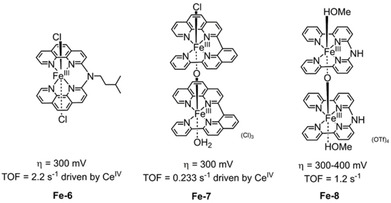
Structure of WOCs **Fe‐6** and **Fe‐7** (pH 1 aqueous buffer),^[42]^ and **Fe‐8** (0.1 M pH 7 Na_2_SO_4_ solution).^[44]^

Two water‐soluble iron‐tetrapyridyl electrocatalysts (**Fe‐6** and **Fe‐7**, Figure 6) were investigated by Thummel et al.^[42]^ Both complexes were shown to be active electrocatalysts in water oxidation, exhibiting similar onset potentials at 1.25 V vs. Ag/AgCl. Cyclic voltammetry at pH 1 showed that the catalytic current of the bimetallic dimer **Fe‐7** was found to be about >6 times larger than the monomeric catalyst **Fe‐6**, mirroring the trends observed in chemical‐driven catalysis using Ce^IV^ affording respective TOF values of 2.2 s^−1^ vs. 0.233 s^−1^. The difference in activity is proposed to result from the formation of a Fe^III^Fe^V^=O dimer intermediate via a disproportionation process in the dinuclear **Fe‐7**. Direct 2e^−^ oxidation of dinuclear **Fe‐7** affords a Fe^IV^Fe^IV^−OH_2_ species, which can undergo a disproportionation to yield the Fe^III^Fe^V^=O species. Monomeric **Fe‐6** requires a greater potential to drive the formation of FeV=O with a 1e^−^ oxidation from Fe^IV^−OH_2_. The advantages of disproportionation in multinuclear complexes is a well‐documented phenomenon, observed for various complexes including Masaoka's pentanuclear Fe catalyst^[11]^ and Meyer's “blue‐dimer” Ru WOC.^[43]^ A similar bimetallic motif ([(MeOH)Fe(Hbbpya)‐μ‐O−(Hbbpya)Fe(MeOH)](OTf)_4_, Hbbpya=*N,N*‐bis(2,2′‐bipyrid‐6‐yl)amine, **Fe‐8**) was later reported by Hetterscheid *et al*., confirming the occurrence of electrocatalytic water oxidation by on‐line electrochemical mass spectrometry. This was reinforced by electrochemical quartz crystal microbalance experiments to rule out the deposition of electrocatalytically active material onto the working electrode.^[44]^ Interestingly, the working electrode material exerts considerable influence on the electrocatalytic performance **Fe‐8**, with a significantly lower overpotential of 300–400 mV with pyrolytic graphite working electrode compared to 600 mV overpotential with a gold working electrode. A similar effect was found for copper WOC **Cu‐7** (vide infra).^[45]^ Therefore, the influence of electrode materials must be considered when attempting to benchmark electrocatalysts.

## 4 Homogeneous Copper‐Based WOCs

As a biologically relevant and earth‐abundant metal, copper has been shown to oxidize phenols, alcohols, and even hydrocarbons due to the availability of high oxidation states of Cu^III^ or even Cu^IV^.^[46]^ With clear mechanism exploration, *d* ^8^ Cu^III^ has been found as an intermediate in reactions of organocopper compounds and in bis(μ‐oxo)‐bridged complexes.^[47]^ Cu^IV^ complexes have been confirmed to be stabilized either by fluoride ligands or as linear O=Cu=O.^[48]^ Additionally, extensive research on copper‐based O_2_‐activating enzymes has been performed, showing facile and reversible O−O bond formation and cleavage under mild conditions.^[46a]^

### 4.1 Copper‐based WOCs in alkaline solution

**Figure 7:**
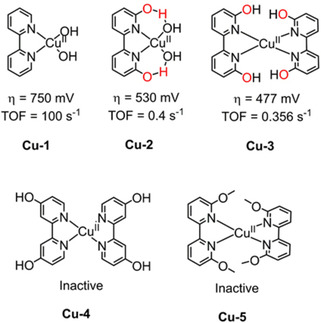
Structures of WOCs **Cu‐1** (0.1 M NaOH/NaOAc at pH 13.1),^[49]^ **Cu‐2** (pH 12.6),^[50]^ and **Cu‐3**, **Cu‐4** and **Cu‐5** (pH 12.6).^[52]^

The first example of a copper WOC (2,2′‐bipyridine)Cu^II^(OH)_2_ (**Cu‐1**, Figure 7) was reported in 2012 by Mayer and they applied it in a 0.1 M aqueous alkaline electrolyte (NaOAc/NaOH, pH 11.8–13.3) buffer solution.^[49]^ The irreversible pH‐dependent oxidation waves at 1.3–1.5 V vs. NHE in alkaline solutions of **Cu‐1** were assigned to water oxidation, as confirmed by irreversible peak at −0.3 V originating from oxygen reduction. The TOF during electrocatalysis was determined to be 100 s^−1^ with an overpotential of more than 750 mV. The resting state of the catalyst was surmised to be the simple monomeric (bpy)Cu(OH)_2_ species through speciation and electrochemical experiments. Importantly, this work opened the door for the use of copper‐based homogeneous catalysts for water oxidation, albeit the large overpotential (>750 mV) and alkaline conditions (pH>11) remain obstacles to be surmounted.

**Figure 8:**
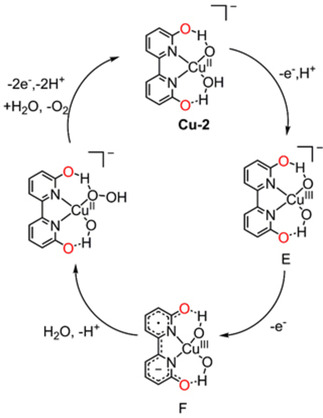
Proposed mechanism of **Cu‐2** for electrochemical water oxidation. (Figure reproduced from ref. [50], Copyright 2014 American Chemical Society, adapted with permission).

In order to improve catalytic efficiency of copper catalysts, Lin et al. developed homogeneous catalysts with pendant 6,6′‐hydroxyl substituted groups (**Cu‐2**, Figure 7) to facilitate proton transport and stabilize the proposed high‐valence intermediates,^[50]^ inspired by the oxygen evolving process in photosystem II, which also utilizes multiple weak interactions.^[51]^ Equimolar amounts of copper(II) salts and 6,6′‐dihydroxybipyridine (6,6′‐dhbp) ligands form a coordination polymer in the solid phase, which catalyzes water oxidation starting at a relatively low overpotential of 510–560 mV in pH 12–14, significantly lower (∼200 mV) than that of (bpy)Cu^II^(OH)_2_ under identical conditions. The reduction in overpotential presumably originates from the non‐innocent ligand 6,6′‐dhbp. Explicitly, the ligand oxidation involved in the oxidation process of intermediate **E** to **F** (Figure 8) enables the generation of oxidizing equivalents to enable water oxidation through a facile Cu^III^ centered intermediate instead of requiring the formation of a harder‐to‐access Cu^IV^ complex, yielding a reduction in overpotential (Figure 8). This work uncovered an effective biomimetic strategy – namely the use of a redox‐active ligands as a tool for the design and preparation of highly efficient WOCs. The application of 6,6′‐dhbp for electrocatalytic water oxidation was revisited by Papish et al. employing a 2 : 1 ligand/copper ratio (**Cu‐3**, Figure 7) in alkaline conditions.^[52]^ For comparison, **Cu‐4** with *exo*‐hydroxy groups and Cu‐5 featuring hydroxy protection were synthesized. CV measurements revealed that **Cu‐3** is active for water oxidation with an overpotential of 477 mV, while **Cu‐4** and **Cu‐5** are inactive under the same conditions. **Cu‐3** demonstrated superior stability and performance over **Cu‐2** under sustained/bulk electrocatalytic conditions. The 2 : 1 ligand/copper ratio effectively suppressed the formation of Cu(OH)_2_ (and other insoluble copper species) in **Cu‐3** solutions, while this is possible for **Cu‐2**, featuring a 1 : 1 ligand/copper ratio.

**Figure 9:**
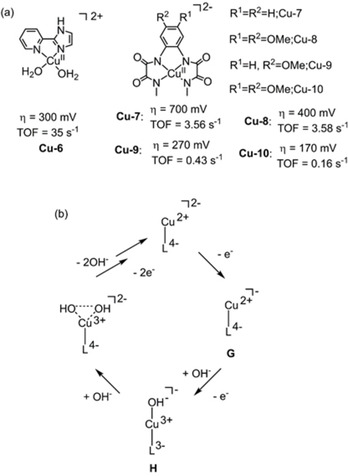
(a) Structures of WOCs **Cu‐6** (0.1 M NaOH/NaOAc at pH 12),^[53]^ and **Cu‐7** to **Cu‐10** (0.1 M phosphate buffer at pH 11.5).^[45]^ (b) The proposed catalytic mechanism of **Cu‐7** to **Cu‐10** for water oxidation.

The use of non‐innocent ligands in Cu‐based WOCs was further explored by Warren et al.,^[53]^ with the introduction of a deprotonatable 2‐(2′‐pyridyl)‐imidazole (pimH) ligand to afford a complex of **Cu‐6** (Figure 9). The ionizable proton of the non‐innocent pimH ligand offers a pathway to a more electron‐rich Cu‐site which is more easily oxidized, leading to an enhancement in catalytic rate (approaching 35 s^−1^) and an overpotential of 300 mV for electrocatalytic water oxidation.

The utility of non‐innocent ligands in improving electrocatalytic water oxidation was exploited by Llobet et al.^[45]^ A family of homogeneous copper WOCs (**Cu‐7** to **Cu‐10**, Figure 9), featuring tetraanionic amidate ligands, *N*_1_,*N*_1_ *′*‐(1,2‐phenylene)bis(*N*_2_‐methyloxalamide) (H_4_L1) was studied at pH 11.5. DFT calculations combined with electrochemical measurements revealed that the RDS during water oxidation electrocatalysis involves the generation of a radical cation species [(L1^.^)Cu^III^(OH)] which reacts with OH^−^, forming an O−O bond, which precedes oxygen evolution (Figure 9b). The behavior of radical species (species **H**, Figure 9b) was strongly influenced by electronic perturbation of the aromatic ring, providing a lever for the control of the thermodynamics and kinetics of the catalytic water oxidation. It was revealed that the increased electron‐donating ligands result in significant decrease in onset potential within this family of copper catalysts. Specifically, with two electron‐donating methoxy groups on the aromatic ring, the overpotential of **Cu‐10** was reduced to 170 mV under basic conditions, while for the complex without methoxy groups (**Cu‐7**) a relatively high overpotential of 700 mV was observed by DPV. Foot‐of‐the‐wave analysis showed that the catalytic rate decreased from 3.56 s^−1^ in **Cu‐7** to 0.16 s^−1^ in **Cu‐10**. A decomposition pathway during water oxidation catalysis was observed, originating from the reactivity of radical cation intermediate **H**. Therefore, these effects need to be carefully balanced in the future design of ligands.

**Figure 10:**
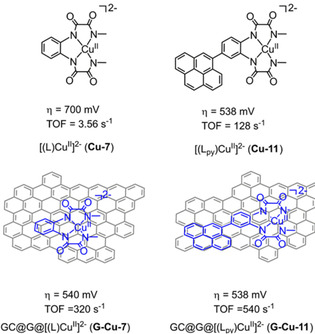
Structural representation of **Cu‐7** and **Cu‐11** and the hybrid materials upon adsorption by π‐stacking to graphitic electrodes (pH 12).^[54]^ (Figure reproduced from ref. [54], Copyright 2017 American Chemical Society, adapted with permission).

Both the onset potential and the rate of catalysis were improved by introduction of pyrene‐functionalized catalyst [(L_py_)Cu^II^]^2−^, (**Cu‐11**, Figure 10, L_py_=4‐pyrenyl‐1,2‐phenylenebis(oxamidate)) improving the π‐delocalization ability of the aromatic ring in the tetraamide moiety of the electrocatalyst.^[54]^ The electrocatalytic performance in water oxidation of **Cu‐7** and **Cu‐11** was performed in solution and compared to the heterogeneous system by modification of graphite electrodes (through π‐π stacking). In homogeneous systems, **Cu‐11** (*η*=538 mV) afforded a ∼160 mV lower overpotential than **Cu‐7** (*η*=700 mV), coupled to a significant increase in *k*_cat_ from 6 to 128 s^−1^ respectively, demonstrating the beneficial effect of an extended π‐system from the electronic perturbation of the pyrene modification. Heterogenization of **Cu‐7** leads to an overpotential of around 540 mV with a *k*_cat_ of 320 s^−1^, benefiting from π‐delocalization provided by graphene. Further enhancements to catalytic performance was achieved upon anchoring **Cu‐11** to a solid support, enabled it to become the most active molecular Cu WOC with an overpotential of 538 mV, a *k*_cat_ of 540 s^−1^, presumably originating from the pyrene moiety and stacking interactions with graphene. Interestingly, for this pyrene appended catalyst the overpotential didn't change upon immobilization on graphene

**Figure 11:**
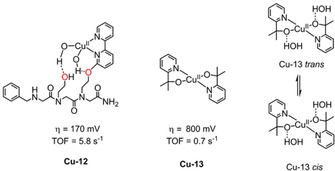
(a) Structures of WOC **Cu‐12** (0.1 m phosphate buffer at pH 11.5)^[55]^ and **Cu‐13** (0.1 m KNO_3_/0.1 m KOH at pH 10.4).^[57]^ (b) *trans*/*cis*‐conversion of **Cu‐13**.^[58]^

In 2018, a copper‐peptido complex **Cu‐12** (Figure 11) was developed to probe the cooperativity between a bis(hydroxy)cuprate metal center and an intramolecular second‐coordination sphere.^[55]^ **Cu‐12** demonstrated electrocatalytic water oxidation, albeit with a high overpotential around 800 mV, but importantly exhibited an impressive stability over a 15 h bulk electrolysis with a Faradaic efficiency of 91 %. Combining DFT calculations, electrochemical experiment and spectroscopic data revealed the high stability originated from intramolecular cooperativity between the active site and hydroxyl group through which the proposed intermediate [LCu^II^(OOH)(OH)] was stabilized. The role of the pendant hydroxyl group as the second coordination sphere tunes catalytic performance and/or stability through the stabilization of intermediates by hydrogen bonding. Alkoxide ligands formed through deprotonation can also bind to metals directly, demonstrated by **Cu‐13**, where alkoxide chelation effectively stabilized the charge of the metal center in high oxidation state intermediates.^[56]^

Following this impetus, a copper WOC of Cu(pyalk)_2_ (**Cu‐13**, pyalk=2‐pyridyl‐2‐propanoate, Figure 11) was found to be active under basic conditions.^[57]^ Electrocatalytic water oxidation with **Cu‐13** required an overpotential of 520–580 mV with a TOF of 0.7 s^−1^. Importantly, it shows a notable stability over 12 h upon bulk electrolysis at 1.1 V vs. NHE. Further mechanistic elucidation revealed that only the *cis* form (Figure 11b) is active for electrocatalytic water oxidation, able to form a metal oxyl radical species, undergoing a water nucleophilic attack (WNA) process, thereby defining the RDS of the reaction.^[58]^

**Figure 12:**
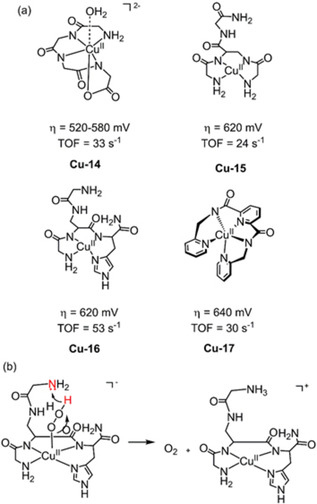
a) Structures of WOC **Cu‐14** (0.25 m phosphate buffer at pH 11),^[59]^ **Cu‐14** and **Cu‐15** (0.15 m phosphate buffer at pH 11),^[60]^ and **Cu‐17** (0.10 m phosphate buffer at pH 8).^[61]^ b) Interaction between H^+^ and glycine in **Cu‐16**.

A well‐defined Cu^II^ complex prepared in situ from the macrocyclic ligand tri(glycyl)glycine ([(TGG_4_^−^)Cu−OH_2_]^2−^, **Cu‐14**, Figure 12), was described as a WOC at pH 11 by Meyer et al. in 2013.^[59]^ A reversible, pH dependent oxidation wave appears at 0.58 V vs. NHE assigned to Cu^III^/Cu^II^ with a 1e^−^/1H^+^ PCET process. At more positive potentials, the robust activity of **Cu‐14** for water oxidation was apparent by an irreversible oxidation wave at 1.32 V exhibiting significant enhancement in the underlying current compared to the blank. Bulk electrolysis of **Cu‐14** showed a constant current density >0.8 mA cm^−2^, over the course of 5 h at 1.3 V vs. NHE and a Faradaic efficiency of 99 %. This combination of impressive activity, stability combined with a facile synthesis makes **Cu‐14** a distinguished WOC. Following this work, the electrochemical behavior for water oxidation of two copper complexes featuring branched peptides **Cu‐15** and **Cu‐16** (Figure 12, featuring ligands H−Gly−Dap(H−Gly)−Gly−NH_2_ and H−Gly−Dap(H−Gly)−His−NH_2_ respectively) was investigated at pH 11 by Malinka et al.^[60]^ Substitution of the glycine in ligand of **Cu‐15** with a proton accepting histidine in **Cu‐16** facilitates PCET pathways in redox processes, leading to an enhancement in TOF (53 vs. 24 s^−1^ with similar overpotential ascribed to the interaction of protons to the pendant lone pair of the amine from the terminal glycine residue (Figure 12b). While the authors did not gain information regarding the nature of key intermediates, the research on the utility of peptides in ligand design offers a potential strategy to lower the overpotential of WOCs.

**Scheme 1:**
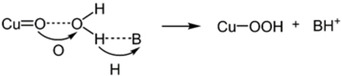
The proposed APT mechanism for electrochemical water oxidation.

As demonstrated the onset potentials of copper‐based WOC can be controlled through facile ligand design.^[45]^ The drive to design oxidatively rugged ligands that (ideally) operate at neutral conditions is evident. With these criteria in mind, the electrochemical properties of a single‐site copper complex Cu^II^(Py_3_P) (**Cu‐17**, Figure 12) was explored in a H_2_PO_4_^−^/HPO_4_^2−^ buffer (pH 8) by Meyer et al.^[61]^ CV of **Cu‐17** exhibits two irreversible anodic waves at 1.29 and 1.5 V vs. NHE, which vary linearly with catalyst concentration suggesting a single‐site mechanism. Furthermore, a linear relationship between the catalytic current and [HPO_4_^2−^] was observed in the range of 0–0.2 M, implying the occurrence on an atom‐proton transfer (APT) process. Shown in Scheme 1, O−O bond formation occurs in concert with proton transfer to the hydrogen‐bonded base. Electrocatalytic water oxidation with an overpotential of 640 mV and catalytic rate of 30 s^−1^ makes **Cu‐17** an excellent candidate for electrochemical water oxidation, considering its operation at a relatively low pH (pH=8) compared to other water oxidation electrocatalysts.

### 4.2 Copper‐based WOCs for electrocatalysis in neutral solution

**Figure 13:**
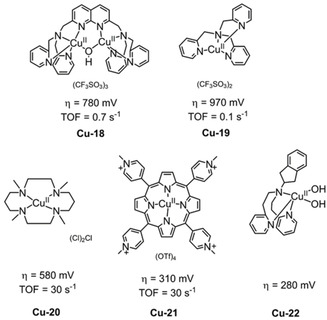
Structures of **Cu‐18**,^[62]^ **Cu‐19**„^[63]^ **Cu‐20**„^[64]^ **Cu‐21**,^[66]^ (0.10 m phosphate buffer at pH 7) and **Cu‐22** ^[67]^ (0.1 m NaNO_3_ at pH 7).

The first example of a homogeneous copper‐based WOC in neutral solutions was reported by Zhang et al.,^[62]^ detailing the electrocatalytic activity of a dinuclear copper complex [Cu_2_(BPMAN)(μ‐OH)]^3+^ (**Cu‐18**, Figure 13, BPMAN=2,7‐[bis(2‐pyridylmethyl)aminomethyl]‐1,8‐naphthyridine) in pH 7 phosphate buffer. CV measurements showed a quasi‐reversible redox wave originating from a Cu^II^/Cu^I^ at *E*_1/2_=0.02 V (vs. NHE) that demonstrated a pH dependence of approximately 59 mV per pH unit, suggesting a 1e^−^/1H^+^ process. Increasing the bias potential to 1.6 V led to an irreversible wave attributed to electrocatalytic water oxidation. Control potential electrolysis (4 h at 1.87 V vs. NHE) in neutral phosphate buffer yielded no evidence of heterogeneity in catalysis implying that the activity stems from a homogeneous complex with an impressive stability of **Cu‐18**. Combining electrochemical insights with DFT calculations revealed that O−O bond formation proceeds via the bimetallic cooperation of two Cu^III^ centers, rather than high‐oxidation state of Cu^IV^=O or Cu^III^/O^<.>^ intermediates reported for monometallic copper WOCs.

The bimetallic catalytic center enabled the reduction of unfavorable electrostatics through cooperatively removing the need for the individual metal centers to achieve high oxidation states (Cu^IV^) during the electrocatalytic cycle. This leads to an enhancement in the rate‐limiting step of O−O bond formation, resulting in the impressive performance of **Cu‐18** at neutral conditions (TOF=0.6 s^−1^). The mononuclear analogue of **Cu‐18**, [Cu^II^(TPA)(OH_2_)]^2+^ (**Cu‐19**, TPA=tris‐(pyridylmethyl)amine, Figure 13) was also investigated by the same group, and found to electrocatalyze water oxidation with larger overpotential of 970 mV and lower TOF of 0.1 s^−1^ via a WNA mechanism where the formal high‐oxidation state of Cu^IV^=O was proposed as an important intermediate.^[67]^ Compared to the dimeric **Cu‐18** catalyst, monomeric **Cu‐19** shows a higher catalytic overpotential by 190 mV and 6 times lower catalytic rate.

Sun et al. reported a single‐site copper complex [Cu^II^(TMC)(H_2_O)](NO_3_)_2_ ^[64]^ (**Cu‐20**, TMC=1,4,8,11‐tetramethyl‐1,4,8,11‐tetraazacyclotetradecane, Figure 13) containing a macrocyclic *N*‐donor ligand with ability to stabilize the metal‐oxo intermediates.^[65]^ In neutral conditions, **Cu‐20** leads a cathodic shift of approximately 200 mV compared to **Cu‐18** in potential to reach a current density of 1 mA cm^−2^.

The water‐soluble tetrakis(4‐*N*‐methylpyridinium) Cu^II^‐porphyrin (**Cu‐21**) was utilized in electrochemical water oxidation under neutral conditions, showing a overpotential for water oxidation of 313 mV at the current density of 0.1 mA cm^−2^ in pH 7 phosphate buffer, dioxygen generation was visually confirmed by observing bubble formation on the FTO working electrode.^[66]^ In pH 3 phosphate buffer, the water oxidation electrocatalysis product switched to H_2_O_2_ from oxygen, measured by rotating ring‐disk electrode voltammetry and formed through a 2e^−^ transfer in the acidic solution. As the formation of peroxide is higher in energy than that of oxygen, this is a relatively unique behavior in WOCs. Another example is [(RPY_2_)Cu(H_2_O)_2_] (**Cu‐22**, RPY_2_=*N*‐substituted bis[2‐pyridyl(ethylamine)] ligands; **R**=indane, Figure 13) which operates at an overpotential of 280 mV at pH 7, the lowest value for water oxidation electrocatalysis by a homogeneous copper‐based catalyst in neutral solution.^[67]^ At pH 6–8, water oxidation was mediated by mononuclear species after the electrochemical oxidation of **Cu‐22**. Analysis of the kinetics of the reaction confirms a mononuclear mechanism for electrocatalytic water oxidation with the RDS being a 1H^+^/2e^−^ process.

## 5 Homogeneous Nickel‐based WOCs

Nickel is an earth‐abundant 1^st^ row TM with Ni^II^, Ni^III^, and Ni^IV^ oxidation states that are easily accessible. Previously a crystal structure of mononuclear Ni^III^‐superoxo complex was reported that demonstrated the potential for nickel catalysts in oxygen activation.^[68]^ The heterogeneous NiO_*x*_ has been confirmed to be an efficient WOC with Ni^IV^ intermediates present in the catalytic cycle.^[69]^ As a result of its readily accessible redox states, it is highly regarded as candidate metal to replace rare elements Ru and Ir in homogeneous water oxidation catalysis.

### 5.1 Nickel‐based WOCs for electrocatalysis in neutral solution

**Figure 14:**
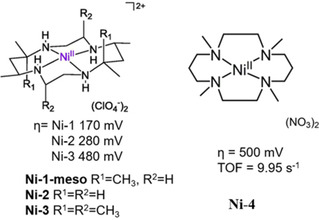
Structures of **Ni‐1**[^70^, ^71^] to **Ni‐4** ^[73]^ (0.10 m phosphate buffer at pH 7).

**Figure 15:**
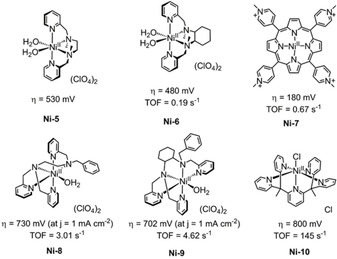
Structures of **Ni‐5** to **Ni‐9** working in neutral conditions,[^74^, ^76^, ^77^] and **Ni‐10** at pH 7–10.8.^[78]^

Lu *et al*. synthesized the first homogeneous nickel WOC in 2014 based on a macrocyclic ligand cyclam ([Ni(*meso*‐Me_6_L)](ClO_4_)_2_ (**Ni‐1**, L=5,5,7,12,12,14‐hexamethyl‐1,4,8,11‐tetraazacyclotetradecane, Figure 14), catalyzing water oxidation with a low overpotential of 170 mV in neutral phosphate buffer.^[70]^ An intramolecular O−O coupling pathway was proposed based on kinetic studies coupled with DFT calculations. Their subsequent study investigated the electrocatalytic activity of analogous tetraazamacrocyclic nickel complexes, varying the degree of methylation on the cyclen ligand (**Ni‐2** and **Ni‐3**, Figure 14) to gain greater mechanistic insights.^[71]^ Computational investigations suggested that a peroxide intermediate was formed through the O−O coupling of two hydroxides coordinated in the same nickel center with phosphate anions acting as proton acceptors. The high catalytic performance (*η*=170 mV at pH 7 for **Ni‐1**) was attributed to the steric effect of the axially oriented methyl groups which suppress inactive Ni^III^‐phosphate species formation. However, work by Dan et al. on the catalytic mechanism of these types of Ni WOCs suggests a greater degree of complexity than that initially proposed by Lu et al., where the role of axial binding of phosphate to the nickel center was neglected.^[72]^ Substitution of the buffer with bicarbonate demonstrated a decrease in redox potential for both the Ni^IV^/Ni^III^ couple and for electrocatalytic water oxidation process, indicating that bicarbonate acts as a non‐innocent axial ligand, oxidized during the redox process. Furthermore, we recently found this catalyst goes through a pH and buffer dependent decomposition pathway: a layer of NiO_*x*_ was formed in a pH 7 phosphate buffer verified by *in situ* characterization of electrochemical quartz crystal microbalance measurements, while no indication of NiO_*x*_ layer formation at a pH of 6.5 in phosphate buffer nor in a pH 7.0 acetate buffer, albeit exhibiting low activity.^[32]^ A similar Ni catalyst with complete *N*‐methylation ([Ni(TMC)(CH_3_CN)](NO_3_)_2_ TMC=1,4,8,11‐tetramethyl‐1,4,8,11‐tetraazacyclotetradecane, **Ni‐4**, Figure 14) was subsequently synthesized by Li et al.^[73]^ It exhibited a moderate overpotential (∼500 mV at pH 7) compared to preceding complexes (**Ni‐1**, **Ni‐2** and **Ni‐3**), but a record TOF (based on Ni) of 9.95 s^−1^ was obtained which is attributed to the increased electron donation properties brought about by methylation of the macrocyclic ligand. Additionally, the catalytic current varies linearly with the proton‐accepting ability (p*K*_a_) of the added base, which plays the important role of regulating catalytic activity through participation in the key O−O bond‐forming step (Scheme 1). Two Ni complexes (NiL−(H_2_O)_2_](ClO_4_)_2_, L=*N,N′*‐dimethyl‐*N*,*N′*‐bis(pyridin‐2‐ylmethyl)‐1,2‐diaminoethane, **Ni‐5** and Ni(mcp)(H_2_O)_2_](ClO_4_)_2_ mcp=(1*R*,2*R*)‐*N*_1_,*N*_2_‐dimethyl‐*N*_1_,*N*_2_‐bis(pyridin‐2‐ylmethyl)cyclohexane, **Ni‐6**, Figure 15) featuring two *cis*‐oriented sites capable of chelating two labile water molecules was investigated by Lu et al.^[74]^ Both complex **Ni‐5** and **Ni‐6** were oxidized directly from Ni^II^ to Ni^IV^ in the catalytic cycle, and demonstrate a moderate overpotential of around 530 mV and 480 mV in sodium acetate buffer (pH 6.5), respectively. Mechanistic investigations suggest that the buffer anion plays an essential role in water oxidation catalysis. Specifically, the PCET‐facilitated water oxidation was expedited by the presence of base that functions as proton acceptor, decreasing the barrier of O−O bond‐formation.

Porphyrins are outstanding ligands, possessing the ability to stabilize high‐valence metal intermediates, and as such have been widely studied for several redox transformations.^[75]^ Cao *et al*. synthesized a water soluble Ni porphyrin WOC ([Ni(Por‐Hpy_4_)]^4+^, Por‐Hpy_4_=*meso*‐tetrakis(4‐*N*‐methylpyridinium)porphyrin, **Ni‐7**, Figure 15) operating across a large pH range (2–8) while maintaining low onset potentials of ∼1.0 V vs. NHE (pH 7) which corresponds to an overpotential of 180 mV only.^[76]^ The electrocatalytic stability of **Ni‐7** was verified by UV‐vis analysis of the solution before and after 10 h of controlled potential electrolysis, with the negligible difference in absorption indicating the homogeneous nature of catalyst. Kinetic investigation of **Ni‐7** revealed a linear relationship between current and catalyst concentration, indicating a single‐site mechanism mediated by [Por^<.>+^−Ni^III^−OH]^5+^.

Two examples of five‐coordinate Ni WOCs featuring a labile water molecule were recently described by Wang et al.^[77]^ **Ni‐8** and **Ni‐9** (Figure 15) electrocatalyzed water oxidation at the similar overpotential of 860 mV in neutral phosphate buffer, while a high rate constants was observed by **Ni‐8** (3.01 vs. 4.62 s^−1^). Although both catalysts have analogous N_5_‐pentadentate ligands, the difference in flexibility arising from the trialkylamine (**Ni‐8**) and diaminocyclohexane (**Ni‐9**) moiety could account for a difference in flexibility that can rationalize the different catalytic performances in electrochemical water oxidation. In addition to high activity, the two complexes exhibited impressive stability and selectivity with a Faradic efficiency of 94–96 % for oxygen production. Another five‐coordinate Ni WOCs **Ni‐10** was reported by Sun et al., which catalyzes water oxidation in an impressive rate of 145 s^−1^ with a relatively large overpotential of 800 mV at pH ranges from 7 to 10.8.^[78]^

### 5.2 Nickel‐based WOCs for electrocatalysis in alkaline solution

**Figure 16:**
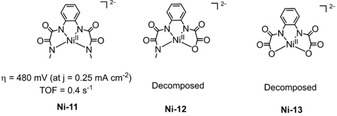
Structures of **Ni‐11**, **Ni‐12**, and **Ni‐13** ^[79]^ (phosphate buffer solution at pH 11).

A family of Ni complexes (**Ni‐11** to **Ni‐13**, Figure 16) with tetradentate ligands were synthesized and studied as electrocatalytic water oxidation catalysts in basic (pH 11) phosphate buffer solution by Ding et al. in 2017.^[79]^ **Ni‐11** was confirmed to act as a homogeneous WOC, while **Ni‐12** and **Ni‐13** merely act as precursors forming heterogeneous materials of NiO_*x*_, as a result of their low electrocatalytic stability. Furthermore, the amount of NiO_*x*_ decomposed on FTO surface by **Ni‐12** is less than that of **Ni‐13**, which is corresponding to the stability order of **Ni‐12**>**Ni‐13**. The results suggest that the presence of an amide donor group is beneficial compared to the carboxylate in ensuring the stability of the nickel WOC.

**Figure 17:**
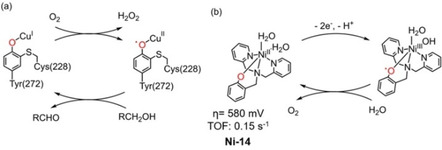
(a) The key rdox‐active species involved in the catalytic cycle of galactose oxidase; (b) the structure of nickel‐phenolate WOC **Ni‐14** ^[81]^ (phosphate buffer solution at pH 11).

Galactose oxidase is a naturally occurring mononuclear‐copper enzyme that catalyzes the two‐electron oxidation of alcohols to aldehydes mediated by a copper(II) tyrosyl radical (Figure 17a).^[80]^ Inspired by this, Wang et al. synthesized Ni WOC **Ni‐14** (Figure 17b), featuring a redox‐active ligand, capable of electrocatalytic water oxidation in neutral phosphate buffer at modest overpotential (400 mV) via the formation of an oxo‐radical intermediate.^[81]^ As illustrated in Figure 17b, Ni^III^L^.^ mediates water oxidation process instead Ni^IV^L by utilizing the phenolate ligand to store and transfer oxidizing equivalents in the electrocatalytic cycle. Just like non‐innocent ligand used in **Cu‐2**,^[50]^ the synergy between the catalytic center metal and an organic radical provides an opportunity to modulate catalyst redox properties to realize improvements in overpotential alongside catalytic activity and stability.

## 6 Homogeneous Cobalt‐Based WOCs

Cobalt as catalytic center for water oxidation has been explored since 1960s.^[82]^ Although these early catalysts show limited activity, they revealed the critical insight that Co^IV^ species was invoked during the catalytic process.^[83]^ Great progress for Co based WOCs has been made by Nocera *et al*. on CoO_x_ species (known as CoPi) that electrodeposited in situ in phosphate buffer at pH 7, which exhibited an overpotential of as low as 400 mV with an excellent stability in the presence of Co^2+^.^[84]^ It is interesting to note that this heterogeneous layer partly dissolves during catalysis as part of the catalytic cycle is in solution. In order to lower the overpotential of homogeneous complexes, many complexes have been designed and explored for electrochemical water oxidation. Some of these cobalt WOCs have been reviewed in the past,^[18a]^ while others were found to act an electrochemical pre‐catalyst – forming CoO_*x*_ during catalytic conditions – which exhibits high activity against electrochemical water oxidation.[^12^, ^25^, ^31^] Therefore in this section, we only focus on examples of stable (i. e. non CoO_x_ forming) electrochemical water oxidation catalysts that operate at neutral conditions.

**Figure 18:**
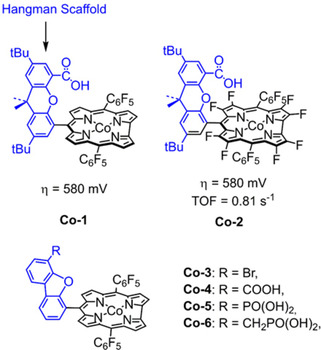
Structures of **Co‐1** ^[85]^ to **Co‐6** ^[87]^ (neutral buffer solution).

**Figure 19:**
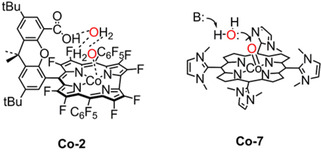
Intramolecular preorganization (**Co‐2**, left) and intermolecular base (**B**:) effect (**Co‐7**, right) facilitating O−O bond formation for structure of **Co‐2** ^[85]^ and **Co‐7**.^[88]^

Two cobalt hangman corroles with β‐octafluoro and *meso*‐pentafluorophenyl substituents (**Co‐1** and **Co‐2**, Figure 18) were confirmed to be electrochemically active (*η*=580 mV and TOF=0.81 s^−1^) for water oxidation in neutral phosphate buffer by Nocera et al.^[85]^ CV measurements revealed that both catalysts show larger currents at lower overpotential than the analogous cobalt catalysts lacking the pendant hangman group, demonstrating the beneficial effect of functional groups that enable in proton coupled electron transfer during electrocatalysis. Subsequent mechanistic investigations into the electrocatalytic mode‐of action for **Co‐1** and **Co‐2**,^[86]^ revealed that the higher electrocatalytic activity originated from the preorganization of water mediated by the hanging group (Figure 19 left) facilitated O−O bond formation. In addition, increasing the p*K*_a_ of the pendant group – an index of proton‐accepting ability – translated to higher activity for electrocatalytic water oxidation. In this regard, the hangman effect was revisited by Cao et al.^[87]^ tuning proton‐accepting ability in cobalt corroles by employing different acid/base pendants including −Br, −COOH, −PO(OH)_2_, −CH_2_PO(OH)_2_. In the line with the hangman effect, the catalytic performance of those cobalt complexes for water oxidation has the order of **Co‐6**>**Co‐5**>**Co‐4**>**Co‐3**, indicating that the RDS of O−O bond formation during the electrocatalytic water oxidation process is enhanced by basic pendant groups that can facilitate proton transfer.

**Figure 20:**
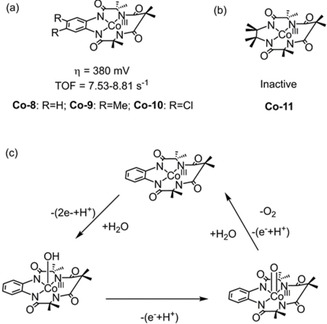
(a) Structures of **Co‐8**, **Co‐9**, **Co‐10** (b) Structure of inactive **Co‐11** and (c) the catalytic cycle of **Co‐8** (neutral buffer solution).^[89]^

A similar base effect in electrocatalytic water oxidation performance was described with homogeneous cationic cobalt porphyrins (Co^II^‐TDMImP, TDMImP=5,10,15,20‐tetrakis‐(1,3‐dimethylimidazolium‐2‐yl) porphyrin, **Co‐7**, Figure 19) as the WOC and the various added buffer anions as proton acceptor by Groves et al.^[88]^ At the same pH of 7, the onset potential and catalytic current varied with p*K*_a_ of buffers (p*K*_a_(HPO_4_^2−^)=7.21, p*K*_a_(phthalate)=5.51, p*K*_a_ (bicarbonate)=6.37, p*K*_a_(*n*‐butylphosphonate)=8.19, and p*K*_a_(*t*‐butylphosphonate)=8.88). The onset potential decreased significantly with increasing basicity of buffer anions; with the lowest value observed for *t*‐butylphosphonate (p*K*_a_=8.88). Mechanistic studies suggest that the high‐valent Co‐porphyrin (Co^IV^−O^.^) intermediate is the reactive oxidant responsible for nucleophilic H_2_O attack. Analogous to the hangman effect, the added base interacts with the proton of a coordinated H_2_O, facilitating O−H bond breaking and subsequent O−O bond formation (Figure 19). Both the dramatic effect of base and the hangman effect illustrate the importance of functional design optimization in both the ligand and the immediate catalytic environment. Recently, a family of Co^III^ WOCs (**Co‐8** to **Co‐10**, Figure 20) based on redox‐active tetraamido macrocyclic ligands (TAML) was investigated at pH 7 by Zhang et al.^[89]^ This class of ligand is appealing for the stabilization of high valence metal ions like Co^IV^ and Fe^V^ that are key intermediates in water oxidation electrocatalysis. For comparison, the TAML analogue with a non‐redox complex **Co‐11** has also been synthesized and studied under the same conditions. The four Co complexes show different electrochemical behaviors strongly correlated to the redox properties of their respective ligands. For **Co‐8**, **Co‐9** and **Co‐10**, CV in phosphate buffer shows two irreversible oxidation processes at 1.00 and 1.47 V vs. NHE. The first oxidation wave was identified as a ligand‐centered PCET oxidation with protons from H_2_O in the buffer, indicating that the ligand is oxidized prior to the chelated Co^III^. The latter oxidation was confirmed to be the electrocatalytic water oxidation process, with the catalytic current varying linearly with catalyst concentration suggesting a single‐site mechanism. Meanwhile, **Co‐11** featuring a non‐redox ligand afforded a non‐catalytic irreversible wave at 1.31 V vs. NHE. A reaction mechanism was proposed (Figure 20c) by combining results from DFT calculations with kinetic analyses. In this catalytic cycle, the mechanism starts with a ligand‐centered (2e^−^/H^+^) PCET oxidation process to form intermediate Co^III^−OH, followed by the formation of the catalytically active intermediate of Co^IV^=O by a subsequent PCET process. This high valent Co^IV^ intermediate combined with water to form a peroxide intermediate that undergoes further oxidation to release dioxygen, and regenerate the catalyst.

## 7 Summary and Outlook

Artificial photosynthesis offers a pathway to the sustainable production of carbon‐neutral fuels by using sunlight as a sustainable energy source. Within this overall conversion process, water oxidation catalysis is key and subject of continues research development, leading to steady improvement of such water oxidation catalysts over the last decade. Water oxidation catalysts based on earth‐abundant 1^st^ row TMs have received an increasing degree of attention in anticipation of accessing scalable catalysts at low cost. In order to achieve this electrocatalytic transformation efficiently catalysts with low overpotential (*η*), that are active (high TOF) and with high durability (high TON) are needed. Several strategies have been taken to improve WOCs on these aspects: 1) introduction of the second coordination sphere to stabilize active intermediates and lower energy barriers; 2) using redox non‐innocent ligands as electron transfer mediators to lower oxidation states of catalytic metal centers, lowering the geometrical changes within a complex upon redox events to increase the rate of electron transfers and thus catalysis; 3) the synthesis of dimeric or multinuclear catalysts to minimize energy of the intermediates and reduce activation barriers; 4) optimizing outer‐sphere environment with added base to lower the barrier in the critical O−O bond formation step.

By exploring or combining the approaches outlined in the above strategies the potential for 1^st^ row TM‐based WOCs to achieve performance comparable to that of catalyst that employ noble metals such as Ru and Ir may be realized. However, challenges remain before practical applications can be realized. It is rare to uncover homogeneous catalysts with all desirable properties – working at low overpotential, under mild conditions with excellent temporal stability – to enable implementation into real‐world prototype devices. For this purpose, further exploration guided by the design rules (vide supra) of WOCs mentioned above serve as a blueprint. Following this course requires deeper mechanistic analyses to be performed, especially the detailed study of intermediates through spectroscopic detection and analysis. It is the advanced chemical analyses of these electrocatalytic systems that will enable a greater understanding of the origins of molecular catalyst deactivation and the intrinsic structural factors that may lead to stability improvements in even better catalysts.

## Conflict of interest

The authors declare no conflict of interest.

## Biographical Information

*Simon Mathew received his Ph.D. in supramolecular photochemistry from Flinders University in 2008. He has conducted research on topics including biofuels, dye‐sensitized solar cells, phototherapeutics, metal‐organic frameworks, gas separation membranes and light‐driven catalysis in Australia, Japan, Switzerland, the U.S and The Netherlands. Currently, he is a laboratory manager in the van't Hoff Institute for Molecular Sciences working with Prof. J. N. H. Reek to develop bioinspired photoelectrocatalytic devices for sustainable chemical transformations and fuel production*.

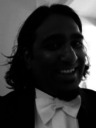

## Biographical Information

*Joost Reek obtained his Ph.D. at the University of Nijmegen under supervision of Prof. R.J.M. Nolte in the area of supramolecular chemistry. After a post‐doc with Prof. Crossley in Sydney, he moved to the University of Amsterdam in 1998, where he was promoted to full professor in 2006, and faculty professor in 2017. His research interests include homogeneous catalysis and supramolecular chemistry, and he is exploring new research on the border of these research topics. In addition, he has an interest in develping solar‐to‐fuel devices based on molecular components, with a focus on the catalytic processes involved*.

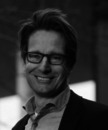

## Biographical Information

*Fengshou Yu received his Ph.D. under the guidance of Prof. Licheng Sun from Dalian University of Technology in 2017. From February 2017 to February 2019, he worked as Postdoctoral Researcher at University of Amsterdam with Prof. Joost N. H. Reek. Since May 2019, he has been working at Hebei University of technology as an independent researcher. Now, he is focusing on developing homogeneous and heterogeneous catalysts for water splitting, CO_2_ reduction and N_2_ reduction*.